# Racial and ethnic differences in comorbid psychosis: a population-based study

**DOI:** 10.3389/fpsyt.2024.1280253

**Published:** 2024-07-29

**Authors:** Mariam Sankoh, James Clifford, Roseann E. Peterson, Elizabeth Prom-Wormley

**Affiliations:** ^1^ Department of Integrative Life Sciences, Virginia Commonwealth University, Richmond, VA, United States; ^2^ Department of Public Health, Brody School of Medicine, East Carolina University, Greenville, NC, United States; ^3^ Department of Psychiatry and Behavioral Sciences, Institute for Genomics in Health, Downstate Health Sciences University, State University of Brooklyn, New York, NY, United States; ^4^ Department of Epidemiology, Virginia Commonwealth University, Richmond, VA, United States

**Keywords:** comorbidity, psychosis, comorbid psychosis, race, ethnicity, population study

## Abstract

**Introduction:**

Differences in the prevalence of psychiatric conditions such as psychosis as well as patterns of comorbidity for psychosis have been reported between racial and ethnic groups. It is unclear whether those differences are consistent for comorbid psychosis

**Methods:**

Self-reported diagnostic data from American adults ages 18–99 participating in the Collaborative Psychiatric Epidemiology Surveys (CPES) (N ~ 11,844) were used to test the association between four racial and ethnic group categories (White, Asian, Hispanic, Black) and comorbid psychosis. Comorbid psychosis was measured as a 4-level categorical variable (No mental illness nor psychosis, Mental Illness, Psychosis only, comorbid psychosis (i.e., Psychosis + Mental Illness). Chi-square tests were used to determine significant differences in the prevalence of comorbid psychosis by race and ethnicity. A multinomial logistic regression was used to test the association between racial and ethnic classifications and comorbid psychosis after adjusting for common demographic characteristics (i.e., education, sex, income, and age).

**Results:**

Relative to White participants, Hispanic and Asian participants were less likely to be affected with comorbid psychosis. (Adjusted Odds Ratio, AOR_Asian_ = 0.32, CI = 0.22 – 0.47, p <0.0001, AOR_Hispanic_ = 0.66, CI = 0.48 – 0.92, p = 0.012). Relative to White participants there was not significant association for comorbid psychosis in Black participants (AOR_Black_ = 0.91, CI = 0.70 – 1.20, p = 0.52) In contrast Hispanic and Black participants were more likely to report psychosis alone (AOR_Hispanic_ = 1.94, CI = 1.27–2.98, p = 0.002, AOR_Black_ = 1.86, 1.24–2.82, p = 0.003) compared to White participants.

**Conclusion:**

There were different patterns of associations by race and ethnicity for psychosis and comorbid psychosis. The lower prevalence of comorbid psychosis in non-White groups may be due to underdiagnosis or underreporting of other mental disorders.

## 1 Introduction

According to the Diagnostic and Statistical Manual of Mental Disorders, Fifth Edition (DSM-5), psychosis is characterized by significant disruptions in thoughts and perceptions, leading to impaired reality testing. Key features of psychosis include hallucinations—perceptions occurring without external stimuli and without insight into their pathological nature—and delusions, which are fixed false beliefs. Psychosis can involve either hallucinations, delusions, or both (1, 2).

In its broadest definition, psychosis represents a profound impairment in reality testing or loss of ego boundaries, significantly interfering with an individual’s ability to meet the ordinary demands of life (2). This condition spans a wide range of psychiatric disorders, including both affective and non-affective disorders. Psychosis is most commonly associated with primary psychotic disorders, where it is a central feature. These include schizophrenia spectrum disorders such as schizophrenia, schizoaffective disorder, delusional disorder, schizophreniform disorder, and brief psychotic disorder (2, 3). However, psychosis is not limited to these conditions. It can also manifest in individuals with bipolar disorder, occurring during manic or depressive episodes, and in those experiencing major depressive episodes as part of major depressive disorder (2, 3). Additionally, psychosis can be a component of secondary psychotic disorders, which are often linked to neurocognitive disorders. These secondary causes include Alzheimer’s disease, Parkinson’s disease, Huntington’s disease, and traumatic brain injuries. In these cases, the psychosis arises as a consequence of the underlying neurological condition (2).

Racial and ethnic disparities in the prevalence of psychosis have been consistently reported in the United States and internationally. Participants who self-identified as Latino (or Hispanic) or Black exhibit a higher lifetime prevalence of psychotic symptoms compared to White or Asian individuals. The lifetime prevalence of self-reported psychotic symptoms in a nationally representative sample of American participants was 15.3% for Black participants, 13.6% for Latino participants, 9.7% for White participants, and 9.6% for Asian participants (4). Moreover, in a clinical sample, it was reported that 27% of people diagnosed with psychosis identified as Black and 17% identified as White (5). These disparities extend beyond the United States, as studies have demonstrated that Black individuals in the United Kingdom have more than twice the relative risk of being diagnosed with psychosis compared to White individuals (6–9).

Comorbid psychosis reflects a substantial proportion of psychosis cases and has been shown to differ significantly by race and ethnicity (10, 11). Comorbid psychosis is defined as a primary psychotic disorder (i.e., schizophrenia, schizoid-affective disorder, or bipolar disorder) that co-occurs with another psychiatric outcome (e.g., generalized anxiety disorder, major depressive disorder, substance use disorders). Approximately 50% of individuals diagnosed with psychosis also experience comorbid psychiatric disorders (12–14). Notably, the specific patterns of comorbidity have been shown to differ by race and ethnicity (15). For instance, among Black Americans with psychosis, the most common comorbid diagnoses are alcohol abuse (27.2%) and drug abuse (20.2%) (15, 16). However, Black patients were significantly less likely to be diagnosed with an affective psychotic disorder, such as, bipolar disorder and major depressive disorder compared to White patients (17, 18).

This study builds on the prior literature by identifying trends of comorbid psychosis across self-identified race and ethnicity groups. We hypothesize that there will be a difference in the prevalence of comorbid psychosis across racial and ethnic groups. Additionally, we expect to find a significant association between comorbid psychosis and race or ethnicity.

## 2 Methods

### 2.1 Study materials and participants

Data from 20,400 participants who completed either a laptop-based or interviewer-administered version of the Collaborative Psychiatric Epidemiology Surveys (CPES) were used. The CPES is a nationally-representative study of adults 18 years and older living in households in the contiguous United States between 2001 and 2003. The CPES encompasses three cross-sectional surveys to ensure participation of people representing a wide range of racial and ethnic experiences: (1) The National Survey on American Life (NSAL), (2) The National Comorbidity Survey Replication (NCS-R), and (3) The National Latino and Asian American Study (NLAAS). Data collection of the CPES were based on the selection of a probability sample of respondents using a multi-stage area probability sampling process of the following population sampling units: (1) US Metropolitan Statistical Areas and counties, (2) area segments, and (3) housing units within the selected area segments. Upon identification of the respondent sample, interviewers contacted housing units in-person conducted a short screening interview with a knowledgeable adult (19). Each participant underwent a validated core interview questionnaire assessing the nature, severity, and impairment of various mental health disorders, including measures of primary mental health diagnostic symptoms, symptom severity, and utilization of mental health services, conducted at different time points (20–23). NSAL, NLAAS, and NCS-R datasets were combined for analysis using a study specific weighting scheme to create combined-analysis weights. Combined analysis weights were created by using the relative size of the study subgroup, defined by race or ethnicity and geographic region.

The original study consisted of 20,400 participants. Participants with incomplete data across any variable used for the statistical analysis were excluded (40.82%). As a result, the final sample size for the analysis was 11,844 participants.

### 2.2 Measures

#### 2.2.1 Psychosis

Psychosis was evaluated in all samples (NSAL, NLAAS, and NCS-R) using the World Health Organization Composite International Diagnostic Interview (WHO-CIDI) psychosis screener, version 3.0 (24). The WHO-CIDI psychosis screener is validated measure derived from the full WHO-CIDI instrument assessing lifetime risk for several health mental disorders (24). Psychosis was developed as a six-level ordinal variable: (1) Ever see visions that others couldn’t see (i.e., visions), (2) Ever hear voices others couldn’t hear (i.e., voices), (3) Ever have a mind control experience (i.e. mind control), (4) Ever feel mind taken over by strange forces (i.e., strange forces), (5) Ever experience communication attempts from strange forces (i.e., communicating w/strange forces), (6) Unjust plots to harm that nobody believes (i.e., plots to harm). Positive endorsement of at least one of these experiences constituted experiencing lifetime psychosis. Respondents in this sample were not excluded when psychosis occurred in the context of falling asleep, dreaming, or substance use.

#### 2.2.2 Mental health disorders

Each mental health disorder was evaluated using the specific WHO-CIDI screener section corresponding to the DSM-IV section Lifetime Mental Health Disorder diagnosis. The following DSM-IV mental health disorders were regrouped into by main disorder type due to low sample sizes in individual disorders: mood disorders (i.e., major depressive episode, dysthymia), anxiety disorders (i.e., post-traumatic stress, generalized anxiety disorder, panic attack, panic disorder, social phobia, agoraphobia, agoraphobia with panic), and substance use disorders (i.e., alcohol abuse, alcohol dependence, drug abuse, drug dependence).

#### 2.2.3 Comorbid psychosis

The outcome variable of comorbid psychosis was developed as a four-level categorical variable: (1) unaffected; (2) psychosis only; (3) mental illness only; and (4) comorbid psychosis (i.e., psychosis and mental illness) and was developed using the corresponding individual items defined according to lifetime mental health disorder section of the DSM-IV. The criteria were evaluated using the WHO-CIDI as validation.

#### 2.2.4 Race and ethnicity

Participants self-identified according to 12 racial or ethnic groups. Participants could only select one category or other if no category representing the race or ethnic group was available. This diverse classification style for race gave participants many options and represented the distribution of commonly identified ethnic groups throughout the United States. Sample sizes in the 12 racial or ethnic groups produced low cell counts (i.e., cell counts less than 5 in some groups) thus were re-classified into four racial or ethnic groups. These four race or ethnicity categories were: Asian (Vietnamese, Filipino, Chinese and All other Asian), Hispanic (Cuban, Puerto Rican, Mexican, and all other Hispanics), Black (Afro-Caribbean and African-American) and White (Non-Latino Hispanic).

#### 2.2.5 Covariates

Self-reported sex was defined as either male or female - a two-level categorical variable. This variable was included because previous research has shown that there may be sex differences in symptomatology in psychosis (25, 26). Psychosis occurs more frequently in men, with a ratio of three diagnosed men to every two diagnosed women, often accompanied by greater disease severity. For example, one study reported that the risk for psychosis was 40% greater in men compared to women (25).

Educational attainment was measured as a continuous variable reflecting the number of years of education. Education was recoded into a four-level categorical variable based on common duration intervals identified in prior literature. The span from 0 to 11 years represents less than high school, 12 years signifies high school, 13 to 15 years represents some college, and 16+ years indicates college graduate and post-college education (27). This structure simplifies educational progression for analysis. Education was included as there is an effect on the onset of psychosis and secondary school completion (27).

Age was measured as a continuous variable. Age was recoded and treated as a four-level categorical variable (i.e., 18–25, 26–45, 46–65, 66+ years old). Age was recoded to assess prevalence estimates by life stage. Age was included since participant age has been associated with psychosis, and prevalence can vary by age (28).

An income-to-needs ratio was used to measure relative poverty and accounts for family size and number of related children younger than 18 years of age (29). The income-to-need ratio was calculated by dividing the reported annual income by the poverty threshold based on the United States Census in 2000, based on when the survey data was collected (30). Continuous ratio values were recoded as a four-level categorical variable (30). This categorical variable was defined relative to poverty (i.e., 0 = poor, 1–2 = near poor, 3+ = non-poor) to account for both socioeconomic status and employment status, similar to prior studies (30). Income was considered because individuals with psychosis struggle with maintaining a certain standard of living in addition to continuous employment (31).

#### 2.2.6 Statistical methods

The subsample of participants included in this study was compared with the remaining participants in CPES who were not included in this study to determine whether the subsample was representative of the CPES sample. Frequencies of a psychosis diagnosis, sex, educational attainment, income-to-needs, and race were compared between the subsample and the remaining CPES participants using a chi-square test.

A chi-square analysis was used to determine significant differences in the prevalence of comorbid psychosis by race or ethnicity. Unadjusted multinomial logistic regression was used to test the association between comorbid psychosis and race or ethnicity. Adjusted multinomial logistic regression was conducted to account for the influence due to the covariates: sex, education level, income-to-needs, and age. All analyses were performed using SAS software, Version 9.4. (SAS Institute Inc, Cary, NC) and accounted for complex survey design and sampling weight using the PROC SURVEYFREQ and PROC SURVEYLOGISTIC.

## 3 Results

### 3.1 Study representativeness

Participants in the subsample exhibited a higher unweighted prevalence of psychosis (4.4%, subsample vs. 0%, non-participants), a higher prevalence of comorbid psychosis (7.1%, subsample vs. 3.2%, non-participants), and higher prevalence of mental illness (34.4%, subsample vs. 9.6%, non-participants) compared to those who did not participate. Additionally, a higher proportion of subsample participants were female (53.3%, subsample vs. 51.6%, non-participants), and attained a college level of education or higher (54.8%, subsample with college education or higher vs. 47.5%, non-participants with college education or higher). A lower proportion were between the ages of 18–45 (51.8%, subsample vs. 57.7%, non-participants). These differences were statistically significant (p < 0.0001). Consequently, the demographic characteristics of the subsample differs compared to that of the full CPES sample. Further, the subsample has a higher prevalence of psychosis, comorbid psychosis and mental illness compared to the full CPES.

### 3.2 Descriptive statistics

Most participants (52.7%) identified as White. Asian participants accounted for 8.2% of the sample, 19.2% of participants identified as Black, and 20.0% of participants identified as Hispanic (**Table 1**). Most participants (78%) completed 12 or more years of education. Similarly, most participants (58.8%) were defined as non-poor. The majority of participants (40.7%) were between the ages of 26–45 (**Table 1**).

**Table 1:** Summary Statistics by racial or ethnic Group (N = 11,844).

	Asian		Black		Hispanic		White		Total	
	N	Weighted %	N	Weighted %	N	Weighted %	N	Weighted %	N	Weighted %
Mental Health Status*										
Unaffected	1502	5.7	2856	10.5	1611	11.6	516	26.4	6485	54.2
Psychosis Only	104	0.5	300	1.2	168	1.2	47	1.5	619	4.4
Mental Illness Only	435	1.7	1511	5.7	893	5.7	950	21.3	3789	34.4
Comorbid Psychosis	75	0.3	444	1.8	268	1.5	164	3.5	951	7.1
Income to Needs *										
Non-Poor	16	0.1	287	1.4	136	1.4	966	24.8	6065	27.7
Near-Poor	38	0.2	263	1.3	168	1.4	2278	64.1	4578	67
Poor	8	0.0	64	0.4	20	0.4	150	4.6	1201	5.4
Educational Attainment*										
Less than High School	9	0.0	233	0.6	159	1.0	760	12.0	1161	13.6
High School	26	0.1	375	1.2	226	1.1	1771	29.1	2398	31.5
Some College	50	0.1	340	0.8	197	0.7	1687	27.3	2274	28.9
College Graduate and Beyond	83	0.2	179	0.4	98	0.4	1692	25.1	2052	26.1
Gender*										
Male	71	0.1	430	1.3	315	1.7	2725	43.7	3541	46.8
Female	97	0.2	697	1.7	365	1.5	3185	49.9	4344	53.3
Age*										
18-25	34	0.1	165	0.5	177	0.9	753	13.7	1129	15.2
26-45	86	0.2	508	1.3	351	1.4	2249	33.7	3194	36.6
46-65	40	0.1	321	0.8	113	0.6	1854	32.2	2328	33.7
>=66	8	0.0	133	0.4	39	0.2	1054	13.9	1234	14.5

*Significant difference by race or ethnicity at p < 0.05.

About 34.4% of all participants reported experiencing mental illness only, 4.4% reported psychosis only, and 7.1% experienced comorbid psychosis (**Table 1**).

Approximately 1.5% of White participants were affected by psychosis only, while 0.5% of Asian and 1.2% of Black as well as Hispanic participants were affected. Approximately 21.3% of White participants, 1.7% of Asian participants, and 5.7% of Black as well as Hispanic participants were affected by mental illness only. Approximately 3.5% of White participants, 0.3% of Asian participants, 1.8% of Black participants, and 1.5% of Hispanic participants were affected by comorbid psychosis.

### 3.3 Unadjusted multinomial logistic region analysis

Compared to White participants, Hispanic participants were 87% more likely to report psychosis only (OR = 1.87, CI = 1.18 – 2.95, p = 0.008) (**Table 2**). Black participants were 95% more likely to report psychosis only (OR = 1.95, CI = 1.30 – 2.96, p = 0.002) compared to White participants. No significant association was detected for Asian participants.

**Table 2:** Summary of Unadjusted Multinomial Associations.

	Psychosis Only vs. Unaffected[ref]	P-value	Mental Illness Only vs. Unaffected[ref]	P-value	Comorbid Psychosis vs. Unaffected[ref]	P-value
	OR (95% CI)		OR (95% CI)		OR (95% CI)	
Race						
White	Reference		Reference		Reference	
Asian	1.47 (0.9 3–2.34)	0.1047	**0.36 (0.30** – **0.44)**	**<.0001**	**0.37 (0.27** – **0.53)**	**<.0001**
Hispanic	**1.87 (1.18 – 2.95)**	**0.0077**	**0.61 (0.52 – 0.73)**	**<.0001**	0.96 (0.73 – 1.25)	0.740`
Black	**1.95 (1.30 – 2.96)**	**0.0017**	**0.67 (0.57 – 0.78)**	**<.0001**	1.25 (0.98 – 1.60)	0.0708
Income to Needs						
Non-Poor	Reference		Reference		Reference	
Near-Poor	**1.74 (1.23 – 2.47)**	**0.002**	0.98 (0.84 – 1.14)	0.8161	**1.64 (1.28 – 2.10)**	**<.0001**
Poor	**1.85 (1.18 – 2.90)**	**0.0076**	1.09 (0.84 – 1.41)	0.532	**1.83 (1.29 – 2.60)**	**0.0008**
Educational Attainment						
Less than High School	1.03 (0.63 – 1.69)	0.9009	**0.69 (0.55 –0.86)**	**0.0011**	**1.72 (1.20 – 2.46)**	**0.0035**
High School	1.02 (0.60 – 1.73)	0.9404	0.86 (0.69 – 1.07)	0.1691	1.37 (0.95 – 1.98)	0.0925
Some College	1.13 (0.69 – 1.85)	0.6229	0.92 (0.74 –1.16)	0.4945	**1.55 (1.07 – 2.25)**	0.0219
College Graduate and Beyond	Reference		Reference		Reference	
Gender						
Male	Reference		Reference		Reference	
Female	1.20 (0.87 – 1.67)	0.2652	**1.25 (1.07 – 1.45)**	**0.0054**	**1.36 (1.08 – 1.72)**	**0.009**
Age						
18-25	1.15 (0.64 – 2.06)	0.6474	**2.35 (1.74 –3.17)**	**<.0001**	**2.52 (1.54 – 4.12)**	**0.0002**
26-45	0.94 (0.55 – 1.60)	0.8206	**2.24 (1.71 –2.94)**	**<.0001**	**2.40 (1.52 – 3.80)**	**0.0002**
46-65	0.74 (0.43 –1.28)	0.1837	**2.56 (1.92 –3.42)**	**<.0001**	**2.57 (1.60 – 4.15)**	**0.0001**
66 and over	Reference		Reference		Reference	

Bold values are statistically significant.

Non-White participants were on average less likely to report having mental illness only compared to White participants (**Table 2**). For example, Asian participants were 64% less likely to report experiencing a mental illness (OR = 0.36, 95% CI = 0.30 – 0.44, p < 0.0001), compared to White participants. Hispanic and Black participants were 39% and 33% less likely to report experiencing a mental illness (OR_Hispanic_ = 0.61, 0.95% CI = 0.52 – 0.73, p < 0.0001; OR_Black_ = 0.67, 95% CI = 0.57 – 0.79, p < 0.0001, Hispanic and Black, respectively), compared to White participants. Similarly, compared to White participants, Asian participants were 63% less likely to report experiencing comorbid psychosis (OR = 0.37, 95% CI = 0.27–0.53, p < 0.0001).

### 3.4 Adjusted multinomial logistic regression analysis

Compared to White participants, Hispanic participants were 94% more likely to report only experiencing psychosis (AOR_Hispanic_ = 1.94, 0.95% CI = 1.2 – 2.98, p = 0.002), and Black participants were 86% more likely to report only experiencing psychosis after accounting for covariates (AOR_Black_ = 1.86, 95% CI = 1.24 – 2.82, p = 0.003) (**Table 3**).

**Table 3:** Summary of Adjusted Multinomial Associations.

	Psychosis Only vs. Unaffected[ref]	P-value	Mental Illness Only vs. Unaffected[ref]	P-value	Comorbid Psychosis vs. Unaffected[ref]	P-value
	OR (95% CI)		OR (95% CI)		OR (95% CI)	
Race						
White	Reference		Reference		Reference	
Asian	1.46 (0.91 – 2.33)	0.1176	**0.30 (0.25 – 0.36)**	**<.0001**	**0.32 (0.22– 0.47)**	**<.0001**
Hispanic	**1.94 (1.27 –2.98)**	**0.0023**	**0.53 (0.44 – 0.65)**	**<.0001**	**0.66 (0.48 – 0.92)**	**0.0123**
Black	**1.86 (1.24 – 2.82)**	**0.0031**	**0.57 (0.48 – 0.68)**	**<.0001**	0.91 (0.70 – 1.20)	0.5173
Income to Needs						
Non-Poor	Reference		Reference		Reference	
Near-Poor	**1.67 (1.11 – 2.53)**	**0.0014**	**1.21 (1.00 – 1.47)**	**0.0049**	**1.60 (1.19 – 2.15)**	**0.0017**
Poor	**1.72 (1.05 – 2.83)**	**0.0325**	**1.35 (1.00 – 1.83)**	**0.0487**	**1.74 (1.14 – 2.66)**	**0.0098**
Educational Attainment						
Less than High School	0.60 (0.35 –1.01)	0.0561	0.77 (0.59 – 1.01)	0.0587	1.50 (0.94 – 2.38)	0.0887
High School	0.76 (0.45 – 1.30)	0.3105	0.84 (0.66 – 1.08)	0.1786	1.17 (0.77 – 1.77)	0.4603
Some College	0.96 (0.58 –1.57)	0.8651	0.87 (0.68 – 1.11)	0.26	1.35 (0.90 – 2.01)	0.1436
College Graduate and Beyond	Reference		Reference		Reference	
Gender						
Male	Reference		Reference		Reference	
Female	1.12 (0.82 – 1.53)	0.4834	**1.24 (1.05 – 1.47)**	**0.0097**	**1.30 (1.02 – 1.64)**	**0.0334**
Age						
18-25	0.86 (0.48 – 1.53)	0.6056	**2.89 (2.10 – 4.00)**	**<.0001**	**2.80 (1.66 – 4.70)**	**0.0001**
26-45	0.76 (0.45 – 1.29)	0.312	**2.76 (2.05 – 3.70)**	**<.0001**	**3.01 (1.84 – 4.93)**	**<.0001**
46-65	0.67 (0.40 – 1.13)	0.1311	**2.91 (2.13 – 3.97)**	**<.0001**	**3.09 (1.88 – 5.08)**	**<.0001**
66 and over	Reference		Reference		Reference	

Bold values are statistically significant.

Compared to White participants, Asian participants were 70% less likely to report experiencing mental illness only (AOR = 0.30, 95% CI = 0.25–0.36, p < 0.001). Hispanics and Black participants were 47% and 43% less likely to report experiencing mental illness only, (AOR_Hispanic_ = 0.53, 95% CI = 0.44–0.65, p < 0.001; AOR_Black_ = 0.57, 95% CI = 0.48–0.68, p < 0.001).

Compared to White participants, Asian participants were 68% less likely to experience comorbid psychosis (AOR_Asian_ = 0.32, 95% CI = 0.22–0.47, p < 0.001). Similarly, Hispanic participants were 34% less likely to experience comorbid psychosis (AOR = 0.66, 95% CI =0.48–0.92, p = 0.001) compared to White participants (**Table 3**).

## 4 Discussion

This is one of the first population-based studies to examine the relationship between comorbid psychosis, psychosis, and mental illness in a United States adult population to include participants that self-identify as non-White race or ethnicity. Three conclusions were identified. First, Black and Hispanic participants were more likely to have a diagnosis of psychosis than White participants. Second, participants identifying as non-White race or ethnicity were less likely to have a diagnosis of a non-psychosis mental illness, compared to participants who identified as White. Third, compared to White participants, Asian and Hispanic participants were less likely to be affected with comorbid psychosis. These results highlight the complexity of the relationship between comorbid psychosis, psychosis, and mental illness among non-White racial or ethnic populations and are discussed below.

### 4.1 Comorbid psychosis in the general population

This study expands on the prior literature by identifying trends of comorbid psychosis by self-identified race or ethnicity. The findings align with our hypothesis that there are discernible differences in prevalence of comorbid psychosis by self-identified race or ethnicity in the general population. Approximately 7.1% of all study participants experienced comorbid psychosis, which is lower than previously reported studies (10, 13, 32). However, these studies focused on clinical or patient-focused studies and often the majority of participants self-identify as White. Therefore, this estimate is likely to be lower because it reflects a prevalence rate for the general population context, rather than patient-based samples.

### 4.2 Differences in comorbid psychosis across racial and ethnic groups

Overall, non-White racial or ethnic participants had a lower prevalence of comorbid psychosis compared to White participants. Black participants (1.8%) had a lower prevalence of comorbid psychosis compared to White participants (3.5%). A similar trend was identified in Hispanic (1.5%) and Asian participants (0.3%). Our results for comorbid psychosis are consistent with many prior studies on comorbid psychosis. However, several studies have yielded varied conclusions concerning comorbid psychosis based on age of onset and accessing and engaging with mental health infrastructure (32–34). Further, multinomial regression results adjusted for the effects of covariates indicated that the odds of being diagnosed with comorbid psychosis was lower for Asian and Hispanic participants compared to White participants. However, this pattern did not extend to Black participants.

It is possible that the detected racial and ethnic differences may reflect measurement non-invariance between racial or ethnic groups. For instance, studies have suggested that symptoms such as paranoia and delusions may be more likely to be labeled as cultural expressions (i.e., creative expression of cultural identity) rather than symptoms of psychosis in certain racial or ethnic groups (35–39). Further, individuals who identify as non-White may be more likely to receive a misdiagnosis or delayed diagnosis of a psychotic disorder due to these measurement differences (40–42). These measurement differences could practically lead to a delay in access to appropriate treatment. Consequently, future studies are encouraged to assess the consistency of measurement non-invariance by race and ethnicity as well as the implications in the epidemiology of these conditions as well as their treatment. These results highlight the need for further research to understand the specific contributions to these disparities.

### 4.3 Psychosis is common in non-white racial or ethnic groups

We were able to reproduce prior results related to a psychosis-only diagnosis in our sample. The odds of being affected with psychosis only in Black and Hispanic participants was almost two times higher than White participants. This result is consistent with previous research, which has also reported higher rates of psychosis among Black and Hispanic participants (4). Moreover, these results align with prior literature that indicates individuals of recent African or Afro-Caribbean descent are more susceptible to experiencing psychosis (8).

Interestingly, despite the higher rates of psychosis diagnosis, Black and Hispanic participants in this study had lower overall rates of mental illness. This paradoxical finding suggests that factors beyond the mere presence of mental illness contributes to the disparities in psychosis rates among different racial and ethnic groups. In particular, the stigma surrounding mental health in certain communities could result in underreporting or underdiagnosing of other mental illnesses among non-White racial or ethnic groups. Consequently, members of some non-White racial or ethnic groups tend to experience higher rates of specific mental health conditions, such as psychosis or substance use, while simultaneously reporting lower overall rates of mental illness when compared to White individuals (43). Avoidance or delay in seeking help could lead to a higher prevalence of severe mental health conditions (e.g., psychosis) when individuals from these communities do eventually access mental health care. The mental health paradox highlights the complexity of mental health disparities and challenges prevailing assumptions about mental health prevalence solely based on racial or ethnic categorizations (43). To address this paradox, it is imperative to conduct more nuanced research that addresses the multifaceted factors influencing mental health outcomes in different communities including income, gender, and age (i.e., birth cohorts). Consequently, these results replicate prior results and highlight the importance of understanding the mental health paradox as it applies to psychosis and comorbid psychosis.

### 4.4 Strengths and limitations

These results should be interpreted while considering the following limitations. First, these data were collected in 2001–2003 and do not capture more recent definitions as detailed in DSM-5, such as changes in duration of symptoms or increased specification across many psychotic disorders. Consequently, these findings may not be generalizable to the current generation of DSM primary psychotic disorders. However, it is a broad definition that may be more applicable to Non-White racial or ethnic communities. Second, the analytic sample size was reduced after removing participants with missing data. However, there were significant differences in the distribution of variables between the full sample and study subsample. The analysis of an imputed sample could result in biased parameter estimates (44), and as such imputation was not conducted. Third, the use of self-reported lifetime prevalence was estimated which may be subject to rater and/or recency bias. However, amongst those who reported experiencing psychosis within their lifetime, 63% had a psychotic experience within the last 12 months. Fourth, participants self-identified their race or ethnicity. Such categorizations are based on socially-derived constructs. Consequently, these results cannot disaggregate the degree to which these results reflect a purely social etiology from social processes that may be subject to genetic confounding (45). Fifth, a significant concern revolves around the potential influence of diverse diagnostic approaches employed across countries and individuals, casting doubt on the generalizability of the results. Psychosis, known for its stability in treatment and diagnosis, is notably consistent, irrespective of cultural constructs (46). The absence of a standardized diagnostic procedure, particularly in distinguishing comorbid psychiatric disorders, raises questions about the transferability of the conclusions to broader contexts. These findings underscore the necessity for additional contextualization of these phenomena by creating more clear diagnosis instructions. Sixth, a critical issue within the study pertains to the diagnostic reliance on the CIDI psychosis screener. While this tool serves as a useful preliminary assessment, it provides only a probable diagnosis rather than a formal one. This limitation introduces an element of uncertainty into the accuracy of the findings. Caution should be exercised when extrapolating implications for clinical practice, although it remains useful when discussing the general population. Furthermore, due to small sample sizes in some individual race and ethnic categories, the study combined certain groups into broader race or ethnicity categories. While this contributed to statistical power of the analysis, it also limited the ability to differentiate how specific groups within the same broader category (e.g., aggregating African Americans and Black Caribbeans in a single Black category) might uniquely experience mental illness.

Despite these limitations, this analysis possesses several significant strengths that contribute to its relevance and value. The study utilizes a large sample that reflects the diversity and complexities of the broader United States population. By incorporating participants from various backgrounds, including immigrant populations and non-White racial or ethnic groups, the research captures a comprehensive picture of mental health experiences across different communities.

## 5 Conclusion

This study highlights the need for continued research and intervention efforts to address the diagnostic gaps and improve mental healthcare provision for non-White racial or ethnic groups with psychosis and comorbid psychiatric disorders. There were notable differences in the risk of psychosis and comorbid psychosis among non-White racial or ethnic groups. Specifically, Hispanic and Asian participants exhibited a significantly reduced likelihood of experiencing comorbid psychosis. Moreover, Hispanic and Black participants were significantly more likely to report experiencing psychosis without comorbid psychiatric disorders. These results suggest a potential disparity in the diagnosis of non-psychosis mental illnesses in non-White racial or ethnic populations.

The observed increased risk for psychosis alone but reduced risk for comorbid psychosis in non-White racial or ethnic groups highlights a potential gap in the recognition and diagnosis of mental health conditions associated with psychosis. It raises concerns regarding biases in how we assess and diagnose comorbid disorders among non-White racial or ethnic groups. To gain a comprehensive understanding of these disparities, a thorough examination of the trends in seeking mental health services and the severity of illness between individuals with psychosis and those with comorbid psychosis is necessary to elucidate the underlying factors contributing to the observed disparities.

Further investigation is warranted to explore help-seeking behaviors, disease severity, and the validity of diagnostic measures across the range of disorders that commonly co-occur with psychosis. Additionally, evaluating the validity of assessment tools for diagnosing comorbid disorders alongside psychosis is crucial to ensure accurate identification and appropriate treatment for individuals within these populations.

In conclusion, comorbid psychosis is a complex mental health condition that requires a multidisciplinary approach to treatment and management, which has not been addressed in our study. It is important to recognize that comorbid psychosis affects individuals from different racial and ethnic backgrounds differently, and that these differences can inform the development of targeted interventions to reduce disparities in diagnosis and treatment. Further research is needed to explore the variation in the diagnosis of psychotic disorders within non-White racial or ethnic groups and to evaluate the measures used to assess comorbid psychosis across different populations. Ultimately, a more nuanced and culturally sensitive approach to the diagnosis and treatment of comorbid psychosis is necessary to ensure that all individuals receive appropriate and effective care. By addressing these challenges, we can strive for more equitable and effective mental health services that appropriately meet the needs of those affected by psychosis and its associated comorbidities.

## Data availability statement

Publicly available datasets were analyzed in this study. This data can be found here: https://www.icpsr.umich.edu/web/ICPSR/studies/20240/summary.

## Ethics statement

The studies involving humans were approved by University of Washington, Cambridge Health Alliance, and the University of Michigan. The studies were conducted in accordance with the local legislation and institutional requirements. Written informed consent for participation was not required from the participants or the participants’ legal guardians/next of kin in accordance with the national legislation and institutional requirements.

## Author contributions

MS: Conceptualization, Data curation, Formal analysis, Methodology, Writing – original draft. JC: Data curation, Writing – review & editing. RP: Writing – review & editing. EP-W: Conceptualization, Funding acquisition, Resources, Writing – review & editing, Methodology, Supervision.
